# Regarding Racial Disparities in Door-to-Clinician Time for Cardiac Chest Pain in the Emergency Department

**DOI:** 10.5811/westjem.63931

**Published:** 2026-06-28

**Authors:** Christian Angelo I. Ventura, Ava D. Cabanilla, Edward E. Denton

**Affiliations:** *Kansas Health Science University, Kansas College of Osteopathic Medicine, Wichita, Kansas; † University of Arkansas for Medical Science, College of Medicine, Little Rock, Arkansas

To the Editor:

Awad and colleagues are to be commended for a methodologically careful examination of racial and ethnic disparities in emergency department door-to-clinician time among a clinically homogeneous cohort of patients with confirmed acute cardiac chest pain.^1^ Their finding that Hispanic/Latino patients experienced a statistically significant 22% increase in door-to-clinician time compared with White patients (18.2 vs 14.9 minutes; adjusted rate ratio 1.22; 95% CI, 1.09–1.36) after adjustment for triage acuity, age, sex, clinician type, and initial vital signs represents a meaningful contribution to the disparities literature. We highlight several considerations that may sharpen the interpretation and translational impact of these results.

First, the authors appropriately acknowledge that their adjusted model did not capture language preference, insurance status, or mode of arrival. We would argue that language discordance between patient and triage clinician may represent an important mechanistic pathway through which the observed disparity operates. Patients with limited English proficiency may require interpreter services at triage, introducing delays that are structurally imposed rather than purely reflective of individual clinician bias.^2^–^4^ Disentangling these mechanisms is not a trivial distinction. If interpreter access is a proximate driver, then one efficient intervention would be universal language screening linked to immediate professional interpreter deployment, rather than implicit bias training alone. The authors’ proposed interventions are reasonable, but future studies should capture language preference and interpreter use directly.

Second, the triage acuity finding deserves closer scrutiny as a potential mediator rather than a covariate. The authors note that Asian patients had the highest proportion of Emergency Severity Index (ESI) level 3 triage (72.9%) and Hispanic/Latino patients were also disproportionately triaged at level 3 (64.0%) relative to White patients (59.3%), a difference that reached statistical significance. If clinician-assigned ESI level is itself shaped by racial or ethnic factors, then adjustment for ESI may partially attenuate a true disparity rather than isolate an independent racial effect.^5^–^7^ A formal mediation analysis partitioning the total effect of race and ethnicity into direct and indirect components through ESI assignment would strengthen causal interpretation and more precisely identify intervention targets within the triage workflow.

Third, the null finding for Black patients warrants careful contextualization. The study enrolled 213 Black patients (5.4% of the cohort) at an institution where non-White patients constitute approximately one quarter of emergency department volume. This sample size affords limited power to detect modest but clinically meaningful differences. The observed adjusted mean of 16.5 minutes for Black patients represents a 1.6-minute excess over White patients, and the confidence interval for the rate ratio (0.94–1.30) remains compatible with disparities of potentially meaningful magnitude. Absence of statistical significance in a small, racially homogeneous sample at a single institution should, therefore, not be taken as definitive evidence of equitable care for Black patients with cardiac chest pain.

Finally, the study period spans 2019 through 2025, encompassing the COVID-19 pandemic and associated disruptions to emergency department workflow, staffing, and patient acuity mix. Without temporal stratification or sensitivity analyses restricted to distinct operational periods, it is difficult to determine whether the observed disparities were stable across this heterogeneous interval or were amplified during periods of greater system strain.

These observations do not diminish the significance of this work. Awad et al provide rigorous single-center evidence that disparities in time-sensitive cardiac evaluation persist despite standardized triage protocols, using a generalized linear model with gamma distribution and log link appropriate for a heavily skewed outcome. Their identification of Hispanic/Latino patients as a particularly vulnerable group in a western U.S. setting that is underrepresented in the disparities literature is an important contribution. We encourage the authors to pursue the multisite replication they advocate, with prospective collection of language preference, interpreter utilization, and operational variables, and to apply mediation frameworks that can move the field from description toward actionable mechanistic understanding.
